# Homozygous A polymorphism of the complement *C1qA*_*276*_ correlates with prolonged overall survival in patients with diffuse large B cell lymphoma treated with R-CHOP

**DOI:** 10.1186/1756-8722-5-51

**Published:** 2012-08-16

**Authors:** Xuan Jin, Huirong Ding, Ning Ding, Zhiying Fu, Yuqin Song, Jun Zhu

**Affiliations:** 1 Department of Internal Medicine Oncology, Peking University First Hospital, Beijing, 100034, China; 2 Key laboratory of Carcinogenesis and Translational Research (Ministry of Education), Central Laboratory, Peking University Cancer Hospital & Institute, Beijing, 100142, China; 3 Key laboratory of Carcinogenesis and Translational Research (Ministry of Education), Department of Lymphoma, Peking University Cancer Hospital & Institute, Beijing, 100142, China

**Keywords:** Complement, Polymorphism, Rituximab, DLBCL, C1qA

## Abstract

**Background:**

The precise mechanism of action for rituximab (R) is not fully elucidated. Besides antibody-dependent cellular cytotoxicity (ADCC), complements may also play an important role in the clinical response to rituximab-based therapy in diffuse large B cell lymphoma (DLBCL). The purpose of this study was to explore the relationship between *C1qA*_*[276]*_ polymorphism and the clinical response to standard frontline treatment with R-CHOP in DLBCL patients.

**Methods:**

Genotyping for *C1qA*_*[276A/G]*_ was done in 164 patients with DLBCL. 129 patients treated with R-CHOP as frontline therapy (R ≥ 4 cycles) were assessable for the efficacy.

**Results:**

Patients with homozygous A were found to have a higher overall response rate than those with heterozygous or homozygous G alleles (97.3% vs. 83.7%,*P* = 0.068). The complete response rate in patients with homozygous A was statistically higher than that in AG and GG allele carriers (89.2% vs. 51.1%,*P* = 0.0001). The overall survival of patients with homozygous A was longer than that of the G allele carriers (676 days vs. 497 days, *P* = 0.023). Multivariate Cox regression analysis showed that *C1qA* A/A allele was an independent favorable prognostic factor for DLBCL patients treated with R-CHOP as first-line therapy.

**Conclusion:**

These results suggest that *C1qA* polymorphism may be a biomarker to predict response to R-CHOP as frontline therapy for DLBCL patients.

## Introduction

Rituximab, a chimeric monoclonal antibody targeted against the pan-B-cell marker CD20, has become a mainstay in the therapy of B-cell non-Hodgkin lymphoma (NHL) [1]. Diffuse large B-cell lymphoma (DLBCL) is the most common lymphoid neoplasm accounting for approximately 30% to 40% of all NHLs. The introduction of rituximab plus cyclophosphamide /doxorubicin /vincristine /prednisone (R-CHOP) chemotherapy has significantly improved the treatment outcome of DLBCL patients, especially non-germinal center B cell-like (non-GCB) subtypes [2-5]. However, not all patients with DLBCL showed good response to R-CHOP chemotherapy. Novel agents and prognostic factors are being explored to improve treatment outcome [6-10].

In addition to synergistic activity with CHOP [11], rituximab also appears to have an antitumor effect itself [12]. Evidences for multiple antitumor mechanisms of rituximab have been reported, including apoptosis [13,14], complement-dependent cytotoxicity (CDC) [15-17], and antibody-dependent cellular cytotoxicity (ADCC) . It has been shown that the antitumor activity of rituximab was completely abolished in syngeneic C1q knockout mice and in a complement-depleted CVF mouse model [18,19]. C1q is the trigger activation of the complement cascade in the presence of immune complexes, it’s formed by six trimers of A, B and C chains. The *C1qA* gene, located on chromosome 1p36.3-p34.1, contains several single nucleotide variations that are currently catalogued in the NCBI database. *C1qA*_*[276]*_ (rs172378) is located at the beginning of the second exon. Racila E et al. reported that among 133 patients with follicular lymphoma treated with single-agent rituximab, polymorphisms in the *C1qA*_*[276]*_ gene may have affected the clinical response and duration of response [20]. However, the impact of *C1qA*_*[276]*_ polymorphism on the efficacy of rituximab in DLBCL patients remains unclear. In this study, we analyzed the relationships between *C1qA* polymorphism and the efficacy of primary R-CHOP therapy in 129 patients with DLBCL.

## Materials and methods

### Patient characteristics and treatment protocol

A total of 164 consented patients who received R-CHOP or R-CHOP-like chemotherapy between June 2007 and December 2010 as a frontline regimen were included in this retrospective study from Beijing Cancer Hospital. All patients had CD20^+^ DLBCL according to the World Health Organization classification as confirmed by our Department of Pathology. Peripheral blood samples from all lymphoma patients were obtained before the initiation of therapy. The clinical research protocol was approved by our Institutional Review Board (IRB).

R-CHOP chemotherapy was administered as follows: one course of chemotherapy consisted of an intravenous infusion of cyclophosphamide 750 mg/m^2^, adriamycin 50 mg/m^2^, vincristine 2 mg, and oral administration of 100 mg prednisone on days 1 to 5, which was repeated every 3 weeks. Rituximab 375 mg/m^2^ was infused over 4 to 6 hours on day 1 before CHOP or CHOP-like chemotherapy was started. Of the 129 patients, 31 patients received radiotherapy in involved-field. The response to R-CHOP therapy was evaluated after completion of 2 to 3 courses of therapy and 1 to 2 months after completion of all planned therapy, then every 3 months for the first year and every 6 months thereafter until progression.

### DNA extraction and genotyping

Genomic DNA was isolated from whole blood with the Whole Blood Genome DNA isolation Kit (spin column) according to the manufacturer’s instructions (Bioteke Corporation, China). DNA was diluted in water to a final stock concentration of 30 ng/ul, and 1ul was used in each PCR reaction. Determination of the *C1qA*_*[276A/G]*_ genotype was achieved blindly on coded specimens by Sanger chain termination sequencing. Briefly, the genomic DNA region of interest was amplified using forward 5’TAAAGGAGACCAGGGGGAAC3’ and reverse 5’TTGAGGAGGAGACGATGGAC3’primers. A first denaturation step at 94°C for 3 min was followed by 35 cycles of denaturation at 94°C for 30 s, annealing at 56°C for 30 s, extension at 72°C for 45 s, and a 10-min final extension step. The PCR products were visualized on a 2% agarose gel and then subjected to direct sequencing.

### Definitions

Patients who had heterozygous (AG) or homozygous G (GG) genotype of *C1qA*_*[276]*_ were designated as G carriers. Clinical responses were determined by physical examination and confirmed by computed tomography or ultrasound. The latter was only used for evaluating superficial lymph nodes. The responses were scored according to International Working Group criteria [21]. Overall survival (OS) was measured from day 1 of the first cycle of R-CHOP until death for any cause or the last follow-up available. The progression-free survival (PFS) was calculated from day 1 of the first cycle of R-CHOP to disease progression or death for any cause.

### Statistical analysis

The clinical characteristics and response rate of the patients were compared using Chi-square, Fisher exact tests according to the C1qA SNP. Kaplan-Meier method was used to estimate the differences of PFS and OS. The Cox regression model was used to evaluate the prognostic factors. Differences between groups were regarded as significant with a p < 0.05. SPSS16.0 was used for all statistical analysis.

## Results

### Patients’ characteristics

The demographics and general characteristics of patients in this study are summarized in Table 1. Enrolled in the study were 81 female and 83 male patients. The median age at diagnosis was 53 years (range, 15–90 years). Eighty nine (54%) patients had stages 3 and 4 disease and 50 (30%) patients had intermediate-to-high or high International Prognostic Index (IPI) scores. Bone marrow was involved by lymphoma in 6 patients (4%) at the time of diagnosis. R-CHOP followed by involved-field radiation was given to 31 (19%) patients. One hundred and twenty-nine patients received R-CHOP as a frontline regimen and were therefore evaluable for this study. A median of 6 cycles of rituximab therapy was given (range, 4–14 cycles), and a median of 6 cycles of chemotherapy was given (range, 2–8 cycles).

**Table 1:** **Patients’ characteristics and their correlations with *C1qA ***_***[276] ***_**genotype**

**Clinical Parameters**	**No.#**	**Genotype**		***P***	**Clinical Parameters**	**No.#**	**Genotype**		***P***
		**AA**	**AG + GG**				**AA**	**AG/GG**	
Gender					Bulky mass				
Male	83	24	59	0.542	≥10 cm	18	5	13	1.000
Female	81	20	61		<10 cm	146	39	107	
Age					Localized				
≤60	102	26	76	0.620	yes	25	7	18	0.886
>60	62	18	44		no	139	37	102	
B symptoms					No Extra Nodal Site				
positive	62	14	48	0.338	≤1	122	31	91	0.484
negative	102	30	72		>1	42	13	29	
LDH					Incidence site				
positive	77	20	57	0.816	lymph node	93	25	68	0.986
negative	87	24	63		extralymph	71	19	52	
β_2_-MG					IPI				
positive	49	10	39	0.203	0~2	114	29	85	0.544
negative	106	32	74		3~5	50	15	33	
Stage					Molecular subtypes				
I~II	75	19	56	0.691	GCB	28	6	22	0.872
III~IV	89	25	39		non-GCB	113	26	87	

Abbreviations: AA /AG/GG indicates C1qA 276 genotype; MG: microglobulin; IPI: international prognostic index; GCB: germinal center B cell.

### *C1qA*_*276*_ polymorphism

The frequency of the *C1qA*_*[276A]*_ allele among all patients with lymphoma was 0.48, whereas the frequency of the *C1qA*_*[276G]*_ allele was 0.52. Forty-four patients (27%) were homozygous A, whereas 50 patients (30%) were homozygous G, and 70 patients (43%) were heterozygous. The genotype distribution of DLBCL population enrolled in our study was in Hardy-Weinberg equilibrium with regard to the *C1qA*_*[276]*_ polymorphism examined (*P* = 0.06).

### Patient characteristics according to *C1qA* allele status

No correlation was observed between patients’ disease features and the *C1qA*_*[276]*_ genotype (Table 1). In the non-GCB subgroup, we observed the associations of the *C1qA*_*[276]*_ genotype with abnormal β2-microglobulin level(defined as level > 3.0) (*P* = 0.012), but not B symptoms (*P* = 0.067). In the GCB subgroup, no association was found between the *C1qA*_*[276]*_ genotype status and any of the factors (Table 2).

**Table 2:** **Prognostic factors and their correlation with *C1qA ***_***[276] ***_**genotype**

**Clinical parameters**	**No. #**	**Genotype**		**χ**^**2**^	***P Value***
Non-GCB					
β_2_-MG		**AA**	**AG + GG**		
positive	35	3	32	6.357	0.012
negative	72	22	50		
B symptoms					
positive	48	7	41	3.344	0.067
negative	65	19	46		
GCB					
β_2_-MG		**AA**	**AG + GG**		
positive	4	2	2		0.218^*^
negative	22	4	18		
B symptoms					
positive	7	3	4		0.144^*^
negative	21	3	18		

Abbreviations: AA /AG/GG indicates C1qA 276 genotype; MG: microglobulin; IPI: international prognostic index; GCB: germinal center B cell.

*:Fisher’s Exact Test.

### Responses to R-chemotherapy according to the *C1qA*_*[276]*_ genotype

Of 129 patients evaluable for response to R-CHOP chemotherapy, the overall response rate (ORR) was 88% (113 of 129 patients), including complete response (CR) 62% (80 of 129 patients), and partial response (PR) rate 26% (33 of 129 patients). As shown in Table 3, homozygous A patients showed a trend of higher response when compared to the G allele carriers (*P* = 0.068). Higher CR rate was observed in patients with homozygous A when compared with G carriers (χ^2^ = 16.263, *P* = 0.0001). Among the responders, CR was seen in 33 of 37 (89.2%) homozygous A, 26 of 55 (47.3%) heterozygous, and 21 of 37 (56.8%) homozygous G patients (Table 3).

**Table 3:** **Response rate according to C1qA **_**276 **_**polymorphism**

**Best response to R-CHOP**	***C1qA***_***[276]***_**genotype**			
	**AA(%)**	**AG(%)**	**GG(%)**	**Total(%)**
Complete response	33 (89.2)	26 (47.3)	21 (56.8)	80 (62.0)
Partial response	3 (8.1)	20 (60.6)	10 (27.0)	33 (25.6)
Overall response	36 (97.3)	46 (83.6)	31 (83.8)	113 (87.6)
Stable disease	0 (0)	0 (0)	0 (0)	0 (0)
Progression	1 (2.7)	9 (16.4)	6 (16.2)	16 (12.4)
Total	37 (100)	55 (100)	37 (100)	129 (100)

Abbreviations: AA /AG/GG indicates C1qA 276 genotype.

In subgroup analysis, a statistically higher CR rate was observed in the homozygous A patients compared with G carriers (81.8% vs 42.9%, χ^2^ = 16.263, *P* = 0.002). This was observed only in patients with non-GCB lymphoma.

### Survival analysis according to *C1qA*_*[276]*_ genotype

**Figure 1:**
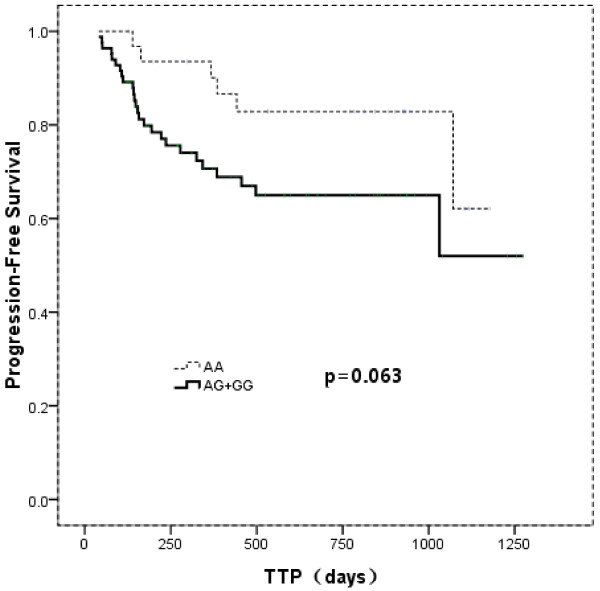
**Kaplan-Meier curve of progression-free survival (PFS) according to *C1qA ***_*** [276] ***_**genotype.**

**Figure 2:**
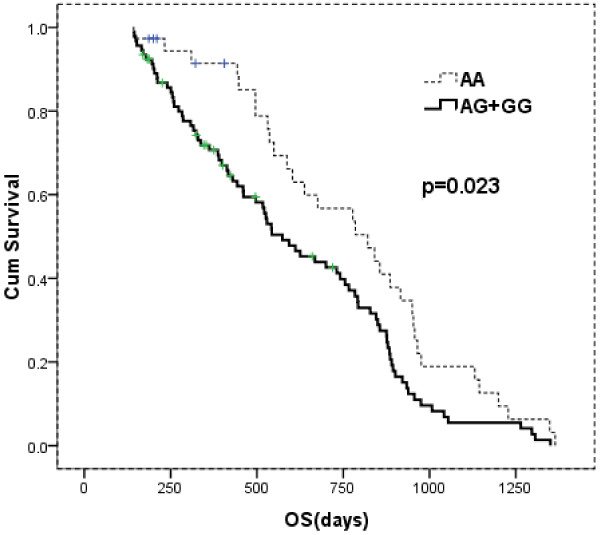
**Kaplan-Meier curve of overall survival (OS) according to *C1qA ***_***[276] ***_**genotype.**

After a median follow-up time of 524 days (range, 60–2073 days), 32 (25%) patients relapsed or progressed, and 18 (14%) died. Seven patients participated in a clinical trial evaluating everolimus (RAD001) and were censored for progression-free survival (PFS) analysis. The homozygous A patients had a median PFS of 623 days (range, 127–1177 days) versus 346 days (range, 42–1255 days) for the rest patients. This longer PFS however did not reach statistical significance (*P* = 0.063) (Figure 1). Survival data was available for 129 patients. The overall survival was 676 days (range, 143–1364 days) for homozygous A patients, and 497 days (range, 142–1350 days) for GG and AG carriers (*P* = 0.023, Figure 2). This difference was not seen when patients were subdivided into GCB- and non-GCB lymphoma groups (data not shown).

### Multivariate analysis

A multivariate analysis was done to evaluate the following variables: stage (stages 1,2 vs. 3,4), IPI score (0–2 vs. 3–5), age (≤60 vs.>60 years), lactate dehydrogenase level (normal vs. abnormal), B symptoms (positive vs. negative), β2-microglobulin level (normal vs. abnormal), extranodal involvement (≤1 site vs. ≥2 sites), bulky mass (≥10 cm vs. <10 cm), and *C1qA*_*[276]*_ (homozygous A vs. G carriers). This analysis confirmed that high IPI score (*P* = 0.033, HR 1.18, 95%CI 1.014-1.375) and abnormal β2-microglobulin level (*P* = 0.001, HR 2.599, 95%CI 1.639-4.121) were poor prognostic factors. When homozygous A was compared with the G carriers for overall survival in this analysis, the relative risk for OS was 0.595 (P = 0.018, 95%CI 0.386-0.915), favoring AA genotype.

## Discussion

In this retrospective analysis, we found that homozygous A -allele carriers of the gene *C1qA*_*[276]*_ had better overall response and higher complete response, and overall survival in DLBCL receiving R-CHOP. In subgroup analysis, higher CR was associated with homozygous A –allele in non-GCB lymphoma, but not GCB type. It is possible that this may be due to the small number of patients that preclude reliable analysis.

As mentioned earlier, a previous study of 133 patients with follicular lymphoma was done on the same C1qA[276][20]. The study showed that in follicular lymphoma patients treated with single agent rituximab, the duration of response and time to progression was significantly prolonged in patients with AA genotype. However, the response rate was similar in both AA and GG genotypes. In our study on DLBCL, the overall response and CR rates were both better in patients with AA genotype of C1qA_[276]_. Since the response was not based on PET/CT scan which is more sensitive than CT alone, the response rate could be different if PET/CT scan were used. PET/CT scan should be considered for response evaluation in future lymphoma studies.

It is unclear exactly how C1qA polymorphism affects the lymphoma responses to R-CHOP chemotherapy. The *C1qA*_*[276]*_ G to A change is a synonymous polymorphism which does not introduce amino acid substitution. It was previously thought that such polymorphisms are “silent”, but now there is a growing body of evidence indicating that synonymous SNPs may alter the expression or function of a protein as well. For instance, synonymous SNPs within the DRD2 transcript can reduce the stability of the mRNA and thus the expression of the dopamine receptor [22]. Another mechanism that could lead to functional effects from a synonymous SNP is biased codon usage [23]. Vasostatin is an NH_2_-terminal fragment of human calreticulin, which is a natural receptor for C1q expressed by many cell types. Pike SE et al. found that vasostatin inhibit angiogenesis and tumor growth [24]. Interaction between calreticulin and C1q inhibits epithelial cell development and angiogenesis [25]. Therefore, C1q activity might affect tumor microenvironment or angiogenesis [20]. Taken all these together, it seems that C1q polymorphism may affect the expression level of C1q, which may account for the differences in clinical responses and treatment outcome in DLBCL. It will be interesting to study the C1q level in lymphoma patients prospectively and see whether it correlates with clinical outcome in lymphoma patients.

## Competing interests

The authors have no conflicts of interests.

## Authors’ contributions

ZJ and SYQ designed the study and review the final manuscript. JX and DHR performed and evaluated the experiment. DN helped to performed the experiment. FZY helped to collect the specimens. JX collected and analyzed data. JX, SYQ and DHR wrote the manuscript. JX and DHR are joint first author, these authors contributed equally to the work. All authors read and approved the final manuscript.
